# Use of point-of-care ultrasound in the analysis of arterial abnormalities in people with chronic kidney disease on hemodialysis: a cross-sectional study

**DOI:** 10.1590/1677-5449.202500362

**Published:** 2026-07-20

**Authors:** Adson Aragão de Araújo Santos, Hellen de Carvalho Lima, Beatriz Vitória Carvalho Lordêlo, Romero Henrique de Almeida Barbosa, Ana Luisa Silveira Vieira, Matheus Rodrigues Lopes, Adirlene Pontes de Oliveira Tenório

**Affiliations:** 1 Universidade Federal do Vale São Francisco – UNIVASF, Paulo Afonso, BA, Brasil.; 2 Faculdade de Medicina de Barbacena – FAME/FUNJOBE, Barbacena, MG, Brasil.

**Keywords:** peripheral arterial disease, ultrasonography, chronic kidney disease, ankle brachial index, hemodialysis

## Abstract

**Background:**

Individuals with chronic kidney disease (CKD) have twice the risk of peripheral arterial disease (PAD) and are more susceptible to arterial calcification. Diagnosing arterial abnormalities involves a combination of clinical assessment and noninvasive tests. The ankle-brachial index (ABI) is a cost-effective method for assessing arterial abnormalities and predicting mortality. Continuous Doppler ultrasound is the gold standard for ABI assessment due to its greater accuracy, and point-of-care ultrasound (POCUS) enables bedside measurement of this index.

**Objectives:**

To analyze the prevalence of arterial abnormalities in individuals undergoing hemodialysis using POCUS.

**Methods:**

We conducted an observational, analytical, cross-sectional study of 85 patients on hemodialysis, using a clinical and sociodemographic questionnaire and calculating the ABI. The primary outcome was the analysis of the presence of arterial abnormalities through the measurement of ABI using the POCUS method..

**Results:**

The prevalence of ABI results suggestive of PAD and arterial calcification was 24.7% and 22.4%, respectively. Diabetes mellitus (p = 0.02) and advanced age (p = 0.01) were identified as the main risk factors for PAD.

**Conclusions:**

The POCUS-assisted assessment identified a prevalence of 24.7% for PAD and 22.4% for arterial calcification among patients with CKD. Advanced age and diabetes mellitus were the main risk factors associated with PAD. The data suggest that POCUS is an innovative approach for the bedside diagnosis of arterial abnormalities in patients on hemodialysis.

## INTRODUCTION

Peripheral arterial disease (PAD) is characterized by arterial involvement of the extremities, especially the lower extremities. PAD results from the obstruction of arterial blood flow due to atherosclerosis, culminating in intermittent claudication, pain, muscle atrophy, hair loss, and thickening of the toenails.^1,2^

PAD affects more than 230 million people, including 10% to 25% of the population over 55 years of age, a risk that increases with age. Smoking and diabetes mellitus (DM) are the main risk factors, followed by hypertension, dyslipidemia, obesity, sedentary lifestyle, history of cardiovascular disease (CVD), male sex, and Black race.^3-6^ PAD significantly impairs quality of life and presents high morbidity and mortality, being considered an independent marker of increased risk of death from CVD.^6^

The risk of PAD is twice as high in individuals with chronic kidney disease (CKD) compared to the general population.^5^ Patients with CKD have a high cardiovascular risk and frequently present with most cardiovascular and PAD risk factors concomitantly.^7,8^ In addition, patients with CKD who undergo hemodialysis are more susceptible to arterial calcification, which is widely distributed throughout the body, especially the lower extremities. The process of arterial calcification is associated with major cardiovascular events and increased mortality in these patients.^9^

A combination of clinical evaluation and noninvasive tests is required to diagnose arterial abnormalities. The initial evaluation should include a detailed medical history, looking for symptoms such as intermittent claudication or leg fatigue. The physical examination should include assessment of the lower extremities, including pallor or capillary refill time.^10^ The ankle-brachial index (ABI) is one of the most cost-effective methods for diagnosing arterial abnormalities, as well as an important predictor of mortality in this at-risk population.^11,12^ This index is considered a primary screening tool, with sensitivity and specificity values > 80%.^13,14^

Approximately 70% of individuals with arterial abnormalities are asymptomatic and, hence, underdiagnosed. Thus, routinely calculating the ABI during physical examinations in general practice becomes even more important.^15,16^ Measuring the ABI in patients at high risk of developing arterial abnormalities is advisable, even in the absence of symptoms.^16^

The ABI is defined as the ratio between the highest systolic blood pressure (SBP) of a lower limb (dorsalis pedis or posterior tibial artery) and the highest SBP of an upper limb (brachial artery). The normal ABI range is considered 0.9 to 1.3. Values ​​≤ 0.9 are associated with PAD, whereas values ​​≥ 1.3 indicate arterial calcification and warrant investigation.^13,17,18^ There are different methods for measuring the ABI. The continuous-wave Doppler method is considered the gold standard because it allows for greater accuracy^19,20^ and is easier to use than stethoscopy for measuring lower limb SBP.^21^

A Doppler-based modality, point-of-care ultrasound (POCUS), is becoming increasingly common in clinical evaluation. This procedure allows for bedside patient assessment and provides a faster, simpler, cheaper, and safer diagnosis than other imaging tests.^22^ POCUS enables visualization of blood vessels and qualitative assessment of arterial flow, which facilitates diagnosis of arterial abnormalities, especially in patients with diabetes who typically have non-compressible arteries due to the calcification process.^23^ POCUS has numerous applications, such as assisting in ABI calculation, which can increase measurement accuracy in various populations. One such population is people undergoing hemodialysis, who generally have mobility restrictions and can benefit from this portable technology. POCUS combines portability, direct anatomical visualization, and dynamic assessment capabilities.^9^

Early detection of arterial abnormalities is essential for preventing ischemic complications. In a recent meta-analysis of 15 studies (9067 arteries), the overall sensitivity and specificity of bedside ultrasound were 0.86 (95% CI 0.81-0.90) and 0.95 (95% CI 0.78-0.97), respectively. Although arteriography is the gold standard for diagnosing PAD, it is an invasive procedure that requires iodinated contrast and exposes patients to ionizing radiation, which limits its routine use in patients with CKD due to the risk of contrast-induced nephropathy.^24^

POCUS has high diagnostic performance for vascular system assessment, with sensitivity ranging from 90% to 97% and specificity up to 93%.^25^ Comparison with traditional screening methods, such as the ABI (69%-79% sensitivity) and the finger-brachial index (83%-85% sensitivity), highlights the technical and diagnostic advantage of POCUS,^11,20,26^ especially in clinical contexts that demand immediate evaluation, such as hemodialysis. Thus, the technique represents an important advance in the early detection and assessment of the severity of vascular changes, favoring more precise and timely clinical interventions.

In the present study, POCUS was used to analyze the prevalence of arterial abnormalities in individuals undergoing hemodialysis.

## METHODS

### Study design

This observational, analytical, cross-sectional study was conducted in accordance with the Strengthening the Reporting of Observational Studies in Epidemiology (STROBE) guidelines. Eligible participants were patients undergoing hemodialysis at the Clínica de Doenças Renais do Vale do São Francisco (CLIRENAL), located in the municipality of Paulo Afonso, Bahia, Brazil.

Because the sample size was calculated based on the finite population of patients undergoing hemodialysis at the clinic (n = 311), the finite population formula was used.^27^ Assuming a 95% confidence level, an expected population prevalence of 28%,^28^ and a maximum tolerated sampling error of 8%, the ideal sample size was calculated at 88 participants. The final sample included 85 individuals, representing virtually all eligible patients at the institution after application of the exclusion criteria.

### Context

In 2022, the city of Paulo Afonso, in the northeastern region of Brazil, had a population of 112,870 inhabitants, of whom 43.6% had a per capita income of ≤ 0.5 times the minimum wage, with 96.4% of all children between 6 and 14 years of age were enrolled in school. The city also had a structured health service network, including all levels of care.^29^

CLIRENAL is the leading provider of hemodialysis and nephrology services in the Paulo Afonso region. As a clinic affiliated with the Brazilian Unified Health System, it receives patients from Paulo Afonso and surrounding municipalities, encompassing the states of Bahia, Alagoas, and Pernambuco.

During the study period, a total of 311 patients underwent hemodialysis at the clinic. These patients, distributed across 3 daily shifts, were subdivided into groups that were treated on alternate days, Monday through Saturday.

### Participants

Patients were invited to participate during hemodialysis treatment, after the study’s objectives had been explained. There was no sampling process (probabilistic or non-probabilistic). Those who met all of the inclusion criteria and none of the exclusion criteria were assessed. However, a minimum sample size was determined to estimate the relationship between the main outcomes, minimizing type II error (false negative).

The participants were selected based on the following inclusion criteria: a confirmed diagnosis of stage 5 CKD (glomerular filtration rate below 15 mL/min/1.73 m^2^) and current conventional hemodialysis. The exclusion criteria were the presence of an arteriovenous fistula and/or lower limb amputation.

**Figure 1:**
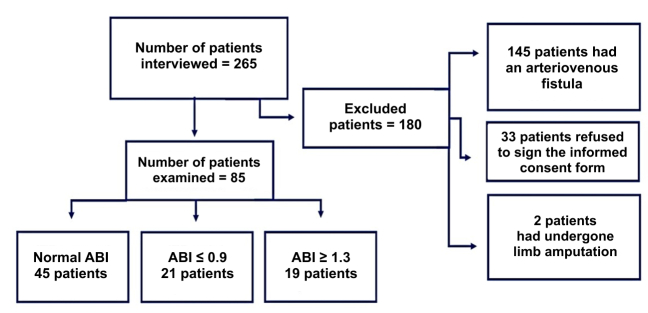
Patient selection flowchart. Caption: ABI = ankle-brachial index.

Clinic visits were conducted during all 3 hemodialysis shifts, from Monday to Saturday, and a clinical and sociodemographic questionnaire was administered to 265 patients on hemodialysis of both sexes, regardless of age or concomitant medical conditions. During subsequent bedside visits with the included patients, POCUS and a physical examination were performed, including palpation of arterial pulses in the lower limbs (pedal and posterior tibial) and blood pressure measurement, which was prioritized during the 20-minute dialysis session to minimize interference with patients’ blood pressure levels. A total of 180 patients were excluded from the study due to the presence of an arteriovenous fistula, lower limb amputation, refusal to participate, or refusal to provide written informed consent (Figure 1).

### Variables

The independent variables were sex, age, BMI, race, duration of dialysis, risk factors (sedentary lifestyle, alcohol use, smoking), comorbidities (hypertension, family history of CVD, DM, CVD, dyslipidemia), and signs and symptoms compatible with PAD.

### Data source

The patients’ clinical and demographic data were collected through medical record review and a semi-structured questionnaire. The questionnaire provided information on age, sex, race, duration of dialysis, presence of vascular access for dialysis, BMI, smoking history, sedentary lifestyle, and family history of CVD.

The following comorbidities were investigated: hypertension, DM, heart disease, stroke, and previous amputations. The presence of signs and symptoms compatible with PAD was characterized by intermittent claudication (muscle pain, mainly in the calves, that appears with exertion and worsens at rest), trophic changes in the extremities (dry and/or cold skin, reduced hair and thickened nails), and non-healing wounds or ulcers.

### ABI calculation

**Figure 2:**
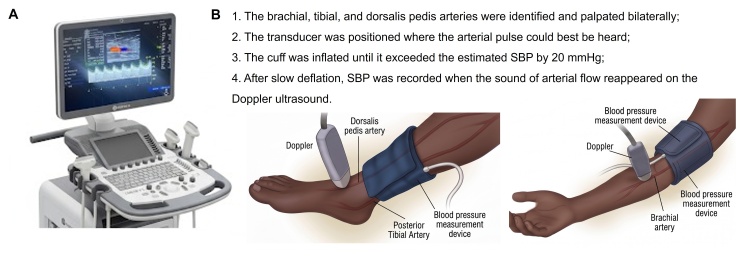
A. Image of the ultrasound device used in the study. B. Steps for measuring systolic blood pressure and obtaining the ankle-brachial index at the bedside. Caption: SBP = systolic blood pressure.

The data for ABI calculation were obtained using a LOGIQ V2 portable ultrasound device in pulsed Doppler mode, with a linear transducer (Figure 2A). The researchers underwent specific prior training to standardize their technique. In cases of dubious results or changes in measurements, the examination was repeated by a more experienced professional to ensure the overall reliability of the data.

SBP values ​​were recorded during the first 20 minutes of the dialysis session, after 5 minutes of rest in a semi-supine position in the hemodialysis chair. The cuff, whose size was appropriate for the arm and ankle circumference, was selected and positioned approximately 2 to 3 cm above the cubital fossa and malleoli, respectively (Figure 2B).

The ABI of the patient’s right and left limbs was then obtained by dividing the highest SBP of the right ankle by the highest SBP of the upper limbs and by dividing the highest SBP of the left ankle by the highest SBP of the upper limbs, respectively.^17,21^ ABI results between 0.9 and 1.3 were considered normal, indicating normal blood flow in the lower limb; results ≥ 1.3 were considered suggestive of arterial calcification; and results ≤ 0.9 indicated arterial stenosis and were considered PAD-related.^13,17^

### Statistical analysis

The data were organized using descriptive statistics and presented in table format. The chi-square test was used to assess the relationship between arterial abnormalities (PAD or arterial calcification) and clinical and sociodemographic variables. Results were considered significant when p < 0.05.

### Ethical aspects

This study was approved by the institutional research ethics committee (certificate: 58588522.3.0000.5666) and was conducted in accordance with the Brazilian National Health Council Resolution 510/2016. CLIRENAL’s authorization and collaboration were also requested.

## RESULTS

A total of 85 patients on hemodialysis were assessed. Of these, 21 (24.7%) had an ABI ≤ 0.9, consistent with PAD (mean: 0.7), and 19 (22.4%) had an ABI ≥ 1.3, consistent with arterial calcification (mean: 1.5).

The prevalence of PAD was higher among women (66.7%, n = 14), those > 60 years of age (76.2%, n = 16), those with a normal BMI (52.4%, n = 11), those who had been on hemodialysis < 12 months (61.9%, n = 13), and those who were sedentary (85.7%, n = 18). The distribution of cases was homogeneous in relation to race (mixed race 38.1% [n = 8], Black 33.3% [n = 7], and White 28.6% [n = 6]).

Arterial calcification was more prevalent among men (63.2%, n = 12), those > 60 years of age (52.6%, n = 10), those with a normal BMI (68.4%, n = 13), those who had been on hemodialysis < 12 months (57.9%, n = 11), and those who were sedentary (78.9%, n = 15). The distribution of cases was homogeneous in relation to race (mixed race 26.3% [n = 5], Black 36.8% [n = 7], and White 36.8% [n = 7]).

Among patients with arterial abnormalities (PAD or arterial calcification), alcohol consumption was more prevalent among those with arterial calcification (63.2%, n = 12), while smoking was more prevalent among those with PAD (57.1%, n = 12). The most prevalent comorbidities were hypertension (> 84.2%) and DM (> 52.6%).

The presence of symptoms, characterized by intermittent claudication, trophic changes in the extremities, and non-healing wounds or ulcers, was higher in the group whose ABI was compatible with PAD (p = 0.03). DM (p = 0.02) and older age (p = 0.01) were associated with a greater chance of PAD (Table 1).

**Table 1:** Clinical and sociodemographic profile of the study participants (N = 85).

**Variables**	**Frequency n (%)**	**ABI n (%)**			
		**Arterial calcification**	**p**	**PAD**	**p**
Female	45 (52.9)	7 (36.8)	0.11	14 (66.7)	0.15
Age > 60 years	45 (52.9)	10 (52.6)	0.66	16 (76.2)	**0.01**
Overweight/obesity	29 (34.1)	6 (31.6)	0.79	10 (47.6)	0.13
Black race	31 (36.5)	7 (36.8)	0.97	7 (33.3)	0.73
Hemodialysis duration > 12 months	31 (36.5)	8 (42.1)	0.56	8 (38.1)	0.86
Sedentary lifestyle	71 (83.5)	15 (78.9)	0.54	18 (85.7)	0.76
Alcohol use	45 (52.9)	12 (63.2)	0.31	9 (42.9)	0.29
Smoking	39 (45.9)	9 (47.4)	0.88	12 (57.1)	0.24
Hypertension	72 (84.7)	16 (84.2)	0.95	19 (90.5)	0.40
Family history of CVD	39 (45.9)	7 (36.8)	0.37	11 (52.4)	0.49
DM	35 (41.2)	10 (52.6)	0.25	13 (61.9)	**0.03**
CVD	32 (37.6)	8 (42.1)	0.65	8 (38.1)	0.96
Dyslipidemia	26 (30.6)	7 (36.8)	0.50	9 (42.9)	0.16
Presence of signs and symptoms consistent with PAD	32 (37.6)	4 (21.1)	0.09	12 (57.1)	**0.03**
Total	85 (100.0)	19 (22.4)		21 (24.7)	

CVD = cardiovascular disease; DM = diabetes mellitus; PAD = peripheral arterial disease; ABI = ankle-brachial index. Statistical analysis performed using the chi-square test.

## DISCUSSION

Arterial abnormalities (PAD or arterial calcification) were found in almost half of our patients on hemodialysis. The main risk factors associated with PAD were DM and advanced age. These findings confirm the importance and need to measure ABI in patients with CKD, due to their susceptibility to cumulative cardiovascular risk factors and the demand for rapid, efficient diagnostic strategies integrated into routine clinical practice.

In our population of patients with CKD, 24.7% had ABIs compatible with PAD. This prevalence is consistent with the findings of a multicenter Spanish study of 2445 patients (28%)^28^ ; however, a Brazilian study found a lower prevalence (11%).^30^

PAD has been described as more prevalent in people with CKD than in the general population.^5^ PAD is a vasculopathy commonly associated with CKD because both share etiological risk factors, and early diagnosis is important to avoid possible complications.^31^ Although signs and symptoms are present in most patients diagnosed with PAD, the clinical manifestations are nonspecific and may originate from musculoskeletal, articular, or venous abnormalities, whether or not they result from CKD. Thus, ultrasound can clarify the etiology. ABI measurement, as a screening tool for PAD, can help minimize adverse outcomes. In this context, technologies such as POCUS can be an important ally in the early diagnosis of PAD and in reducing its complications.^32-34^

Most of our patients with PAD were smokers. Although smoking is a significant risk factor for PAD, we found no significant association with the disease, probably due to the limited number of patients who participated in the study.

DM was associated with PAD, corroborating previous studies.^6,28,30^ This reaffirms the important influence of DM on the genesis of PAD, since DM produces vascular inflammation and epithelial damage, in addition to vasoconstriction associated with platelet activation.^35^ Although other comorbidities, such as hypertension, obesity, cerebrovascular disease, and dyslipidemia, are also risk factors for PAD, no significant association with them was found in our sample.

Because hemodialysis causes long-term vascular changes,^36^ individuals who undergo this treatment for a longer period are expected to have a higher prevalence of PAD. However, no association was observed between dialysis duration and PAD incidence, a finding also reported in previous studies.^30,37^

In 22.4% of examinations, an ABI ≥ 1.3 was observed, consistent with arterial calcification. In previous research, Ramos et al.^30^ reported that up to 46.2% of patients undergoing hemodialysis had vascular calcification. This difference may be due to the duration of patients’ hemodialysis.^38^ Arterial calcification, a significant complication of CKD, results from a disturbance in mineral and bone balance and confers a higher risk of mortality in affected individuals.^38,39^

Arterial calcification is related to increased vascular stiffness and the presence of non-compressible arteries. This leads to greater thickening of the renal arteries, a higher prevalence of renal microvascular injury, and consequently, a reduced glomerular filtration rate. Furthermore, a high ABI, for example, can lead to hemodynamic overload, endothelial dysfunction, and left ventricular hypertrophy, increasing the risk of cardiovascular events, especially in patients with CKD.^40^ Just as with PAD, arterial calcification can be clinically investigated by measuring the ABI.

Through visualization of the artery, POCUS facilitates SBP measurement and enables ABI calculation. This is even more important in patients who routinely present with arterial abnormalities (such as calcification) and are evaluated in collective settings, at the bedside, or in noisy environments. Furthermore, POCUS allows trained professionals to more comprehensively assess PAD, facilitating disease stratification and better case management. As has been highlighted by other authors, POCUS is also useful for assessing other important organs and systems in the context of CKD and hemodialysis.^21,22,34^

It should be noted that the POCUS method has certain limitations, as it requires experience, specialized training, and time to implement in large-scale practice.^32,34^ One study limitation was that ABI values ​​≥ 1.3 may underestimate the prevalence of PAD, since false-negative test results can occur in this population. Another limitation concerns the large proportion of patients with arteriovenous fistulas as vascular access for dialysis, which limited the study population and the final number of participants in each group, preventing more robust statistical analyses.

Because this was an observational, cross-sectional study, cause-and-effect relationships between risk factors and arterial abnormalities could not be determined. Furthermore, the study was conducted at a single center, which limits the generalizability of the results to other hemodialysis populations or to different geographic and health contexts.

## CONCLUSIONS

Using POCUS, we identified a prevalence of 24.7% for arterial abnormalities compatible with PAD and 22.4% for those compatible with arterial calcification in our sample of patients with CKD on hemodialysis. Moreover, advanced age and DM were the main factors associated with PAD in these individuals. POCUS proved a feasible method for measuring ABI and detecting arterial abnormalities at the bedside at our study center, reinforcing the need for continuous vascular monitoring in this population, as vascular impairment was found in almost half of the participants.
