# Supplementary material for: Use of point-of-care ultrasound in the analysis of arterial abnormalities in people with chronic kidney disease on hemodialysis: a cross-sectional study
Source: J Vasc Bras. 2026 Jul 20;25:e20250036. doi: 10.1590/1677-5449.202500362 (PMC13441215; doi:10.1590/1677-5449.202500362)
Supplement: Translation

# Translation: Utilização de ultrassonografia *point-of-care* na análise de alterações arteriais em pessoas com doença renal crônica em hemodiálise: estudo transversal

**DOI:** 10.1590/1677-5449.202500361

**Authors:** Adson Aragão de Araújo Santos, Hellen de Carvalho Lima, Beatriz Vitória Carvalho Lordêlo, Romero Henrique de Almeida Barbosa, Ana Luisa Silveira Vieira, Matheus Rodrigues Lopes, Adirlene Pontes de Oliveira Tenório

**Affiliations:** 1 Universidade Federal do Vale São Francisco – UNIVASF, Paulo Afonso, BA, Brasil.; 2 Faculdade de Medicina de Barbacena – FAME/FUNJOBE, Barbacena, MG, Brasil.

**Keywords:** doença arterial periférica, ultrassonografia, doença renal crônica, índice tornozelo-braço, hemodiálise

## Abstract

**Contexto:**

Indivíduos com doença renal crônica (DRC) apresentam risco duas vezes maior de doença arterial periférica (DAP) e são mais suscetíveis a calcificação arterial. O diagnóstico das alterações arteriais envolve a combinação de avaliação clínica e testes não invasivos. O índice tornozelo-braquial (ITB) é um método de avaliação de alterações arteriais, preditivo de mortalidade, com bom custo-benefício. O método Doppler contínuo é considerado padrão-ouro para análise de ITB, pois proporciona maior precisão, e a ultrassonografia *point-of-care* (POCUS) permite obter essa medida à beira leito.

**Objetivos:**

Analisar a prevalência de alterações arteriais em indivíduos em hemodiálise por meio da POCUS.

**Métodos:**

Estudo transversal, analítico e observacional realizado com 85 pacientes em hemodiálise, por meio da aplicação de questionário clínico e sociodemográfico e cálculo do ITB. O desfecho do estudo consistiu na análise da presença de alterações arteriais por meio da mensuração do ITB por meio do método POCUS.

**Resultados:**

Evidenciou-se prevalência de 24,7% e 22,4% de pacientes com ITB sugestivo de DAP e de calcificação arterial, respectivamente. Diabetes melito (p = 0,02) e idade avançada (p = 0,01) foram identificados como os principais fatores de risco para DAP.

**Conclusões:**

A avaliação com auxílio da POCUS identificou prevalência de 24,7% para DAP e 22,4% para calcificação arterial entre os pacientes com DRC. Idade avançada e diagnóstico de diabetes melito foram os principais fatores de risco associados à DAP. Os dados indicam que a POCUS é uma abordagem inovadora para diagnosticar alterações arteriais à beira leito em pacientes em hemodiálise.

## INTRODUÇÃO

A doença arterial periférica (DAP) caracteriza-se pelo acometimento arterial das extremidades, especialmente dos membros inferiores. A DAP decorre da obstrução do fluxo sanguíneo arterial devido à aterosclerose, o que culmina em claudicação intermitente, dor, atrofia muscular, perda de pelos e espessamento das unhas do pé^1,2^ .

A DAP atinge mais de 230 milhões de pessoas e acomete entre 10 e 25% da população com mais de 55 anos, risco que aumenta com a idade. Tabagismo e diabetes melito (DM) são os principais fatores de risco, acompanhados por hipertensão arterial, dislipidemia, obesidade, sedentarismo, histórico de doença cardiovascular (DCV), sexo masculino e etnia negra^3-6^ . A DAP acarreta comprometimento considerável da qualidade de vida e apresenta morbimortalidade elevada, sendo considerada um marcador independente de risco aumentado de morte por DCV^6^ .

Indivíduos com doença renal crônica (DRC) têm risco duas vezes maior de DAP em comparação com a população geral^5^ . Pacientes com DRC possuem elevado risco cardiovascular e frequentemente apresentam a maioria dos fatores de risco cardiovascular e de DAP concomitantemente^7,8^ . Além disso, pacientes com DRC em hemodiálise estão mais suscetíveis a calcificação arterial, amplamente distribuída por todo o corpo, especialmente nos membros inferiores. O processo de calcificação arterial está associado a eventos cardiovasculares maiores e ao aumento da mortalidade nesses pacientes^9^ .

O diagnóstico das alterações arteriais envolve a combinação de avaliação clínica e testes não invasivos. A avaliação inicial deve incluir realização de coleta detalhada da história clínica, que busca sintomas como claudicação intermitente ou fadiga nas pernas. O exame físico deve incluir avaliação das extremidades inferiores, palidez ou tempo de enchimento capilar^10^ . A mensuração do índice tornozelo-braquial (ITB) é um dos métodos com melhor custo-benefício para diagnóstico de alterações arteriais, além de um importante preditor de mortalidade nessa população de risco^11,12^ . Esse índice é considerado ferramenta de triagem primária e seus valores apresentam sensibilidade e especificidade superiores a 80%^13,14^ .

Aproximadamente 70% dos indivíduos com alterações arteriais são assintomáticos e, por isso, subdiagnosticados. A realização do cálculo do ITB de rotina durante o exame físico na prática clínica de médicos generalistas se torna ainda mais importante^15,16^ . Dessa forma, a mensuração do ITB em pacientes com elevado risco de desenvolver alterações arteriais passa a ser indicada, mesmo na ausência de sintomas^16^ .

O ITB é definido como a razão entre a maior pressão arterial sistólica (PAS) de um membro inferior (artéria pediosa dorsal ou tibial posterior) e a maior PAS de um membro superior (artéria braquial). Considera-se normal o intervalo do ITB entre 0,9 e 1,3. Valores ≤ 0,9 são associados à DAP e ≥ 1,3 indicam que deve ser realizada investigação de calcificação arterial^13,17,18^ . Existem diferentes métodos de mensuração do ITB. O método Doppler contínuo é considerado padrão-ouro para análise do ITB, pois permite maior precisão^19,20^ , além de ser considerado mais fácil de utilizar do que o estetoscópio na mensuração da PAS nos membros inferiores^21^ .

Uma das modalidades baseadas no Doppler, a ultrassonografia *point-of-care* (POCUS), tem sido cada vez mais utilizada na avaliação clínica dos pacientes. Esse procedimento possibilita a avaliação dos pacientes à beira leito e proporciona um diagnóstico mais rápido, simples, barato e seguro do que outros exames de imagem^22^ . A utilização da POCUS permite a visualização de vasos sanguíneos e a avaliação qualitativa do fluxo arterial, o que auxilia no direcionamento diagnóstico de alterações arteriais, especialmente em pacientes com diabetes, que normalmente apresentam artérias não compressíveis em virtude do processo de calcificação^23^ . A POCUS tem inúmeras aplicabilidades, como auxiliar na determinação do ITB, o que pode aumentar a acurácia da mensuração em diferentes populações. Entre esses, estão as pessoas em hemodiálise, que geralmente têm restrições de mobilidade e podem se beneficiar do uso dessa tecnologia portátil. A POCUS associa portabilidade, visualização anatômica direta e capacidade de avaliação dinâmica^9^ .

A detecção precoce de alterações arteriais é essencial para a prevenção de complicações isquêmicas. Em uma metanálise recente, que incluiu 15 estudos e avaliou 9.067 artérias, a ultrassonografia à beira leito demonstrou sensibilidade global de 0,86 [intervalo de confiança de 95% (IC95%) 0,81-0,90] e especificidade de 0,95 (IC95% 0,78-0,97). A arteriografia é considerada padrão-ouro para o diagnóstico de DAP; no entanto, trata-se de um método invasivo que requer o uso de contraste iodado e exposição à radiação ionizante, o que limita sua aplicação rotineira em pacientes com DRC devido ao risco de nefropatia induzida por contraste^24^ .

A POCUS demonstra elevado desempenho diagnóstico na avaliação do sistema vascular, caracterizando-se por sensibilidade entre 90 e 97% e especificidade de até 93%^25^ . A comparação com métodos tradicionais de triagem, como o ITB (sensibilidade entre 69 e 79%) e o índice dedo-braquial (sensibilidade entre 83 e 85%), evidencia a vantagem técnica e diagnóstica da POCUS^11,20,26^ , sobretudo em contextos clínicos que demandam avaliação imediata, como na hemodiálise. Dessa forma, sua utilização representa um avanço importante na detecção precoce e na avaliação da gravidade das alterações vasculares, favorecendo intervenções mais precisas e oportunas na prática clínica.

Assim, este estudo propõe analisar, por meio da POCUS, a prevalência de alterações arteriais em indivíduos em hemodiálise.

## MÉTODOS

### Desenho do estudo

Trata-se de um estudo transversal, analítico e observacional, realizado conforme as diretrizes do *Strengthening the Reporting of Observational Studies in Epidemiology* (STROBE) *Statement*. Os participantes deste estudo foram pacientes em tratamento hemodialítico na Clínica de Doenças Renais do Vale do São Francisco (CLIRENAL), localizada no município de Paulo Afonso, BA, Brasil.

O dimensionamento amostral foi calculado com base na população finita de pacientes em hemodiálise na clínica estudada (n = 311). Utilizou-se a fórmula para populações finitas^27^ , adotando-se nível de confiança de 95%, prevalência populacional esperada de 28%^28^ e erro amostral máximo tolerado de 8%. O cálculo indicou um tamanho amostral ideal de 88 participantes. A amostra final do estudo incluiu 85 indivíduos, o que representa praticamente a totalidade dos pacientes elegíveis na instituição, após a aplicação dos critérios metodológicos de exclusão.

### Contexto

Paulo Afonso situa-se na região Nordeste do Brasil, no sertão baiano, e em 2022 contava com uma população de 112.870 habitantes, da qual 43,6% apresentavam rendimento per capita de até 0,5 salário-mínimo, com 96,4% de taxa de escolarização entre 6 e 14 anos de idade. A cidade também contava com uma rede de serviços de saúde estruturada com todos os níveis de atenção^29^ .

A CLIRENAL é a principal empresa de fornecimento de terapia hemodialítica e serviços de nefrologia da região de Paulo Afonso. Por ser uma clínica vinculada ao Sistema Único de Saúde, recebe pacientes do município de Paulo Afonso e dos demais municípios circundantes, abrangendo os estados da Bahia, Alagoas e Pernambuco.

Durante o período contemplado no estudo, um total de 311 pacientes estava em terapia hemodialítica na clínica. Esses eram distribuídos em três turnos diários e subdivididos em grupos que compareciam à clínica em dias alternados, de segunda a sábado.

### Participantes

O convite para participação foi realizado durante o tratamento hemodialítico, após a explicação do objetivo da pesquisa. Não houve processo de amostragem (probabilística ou não probabilística). Indivíduos que contemplavam os critérios de inclusão e não apresentavam critérios de exclusão foram avaliados. No entanto, o tamanho mínimo de dados foi dimensionado para estimar a relação entre os principais desfechos, minimizando o erro do tipo II (falso-negativo).

Os entrevistados foram selecionados com base nos seguintes critérios de inclusão: possuir diagnóstico confirmado de DRC estágio 5 (taxa de filtração glomerular abaixo de 15 mL/min/1,73 m^2^) e estar em tratamento hemodialítico convencional. Os critérios de exclusão foram a presença de fístula arteriovenosa e/ou amputação de membro inferior.

Foram realizadas visitas à clínica nos três turnos de sessão de hemodiálise, de segunda a sábado, e foi aplicado o questionário clínico e sociodemográfico a 265 pacientes em hemodiálise, de ambos os sexos, independentemente da idade e da presença de outras condições médicas concomitantes. Posteriormente, para aqueles incluídos no estudo, foram realizadas visitas à beira leito para a realização da POCUS, bem como o exame físico com palpação de pulsos arteriais de membros inferiores (pediosos e tibiais posteriores) e aferição da pressão arterial, prezando pela realização dessa abordagem durante os 20 minutos de diálise para minimizar a interferência desse procedimento nos níveis pressóricos dos pacientes. Foram excluídos do estudo 180 pacientes devido à presença de fístula arteriovenosa, amputação de membro inferior e recusa a participar ou assinar o Termo de Consentimento Livre e Esclarecido (Figura 1).

**Figura 1:** Fluxograma de seleção dos pacientes. Legenda: ITB = índice tornozelo-braquial; TCLE = Termo de Consentimento Livre e Esclarecido.

### Variáveis

As variáveis independentes foram sexo, idade, índice de massa corporal (IMC), etnia, tempo de diálise, presença de fatores de risco (sedentarismo, uso de álcool, tabagismo), presença de comorbidades (hipertensão arterial, histórico familiar de DCV, DM, DCV, dislipidemia) e presença de sinais e sintomas compatíveis com DAP.

### Fonte de dados

Os dados clínicos e demográficos dos pacientes foram coletados por meio da avaliação de prontuários e de questionário semiestruturado. Com o questionário, foi possível obter informações sobre idade, sexo, etnia, tempo de diálise, presença de acesso vascular para diálise, IMC, histórico de tabagismo, sedentarismo e histórico de DCV na família.

Acerca das comorbidades, investigou-se a presença de hipertensão arterial, DM, cardiopatias, acidente vascular encefálico e amputações prévias. A presença de sinais e sintomas compatíveis com DAP foi caracterizada por claudicação intermitente (dor muscular, principalmente nas panturrilhas, que surge com o esforço e piora no repouso), alterações tróficas em extremidades (pele seca e/ou fria, redução de pelos e unhas espessas) e feridas ou úlceras que não cicatrizavam.

### Cálculo do ITB

As informações para o cálculo do ITB foram obtidas com um ultrassom portátil, modelo LOGIQ V2, no modo Doppler pulsátil, utilizando o transdutor linear (Figura 2A). Os pesquisadores passaram por um treinamento prévio específico para padronização da técnica. Em casos de resultados duvidosos ou alteração na aferição, o exame era repetido por outro profissional com maior experiência no método, de forma a garantir total confiabilidade dos dados.

**Figura 2:** A. Representação do aparelho utilizado no estudo. B. Etapas para aferição da pressão arterial sistólica e obtenção do índice tornozelo-braquial à beira leito. Legenda: PAS = pressão arterial sistólica.

Os valores da PAS foram obtidos e registrados após 5 minutos de repouso na posição semi-supina, na poltrona de hemodiálise, nos primeiros 20 minutos do procedimento. O manguito, com tamanho adequado às circunferências do braço e do tornozelo, foi selecionado e posicionado cerca de 2 a 3 cm acima da fossa cubital e dos maléolos, respectivamente (Figura 2B).

Por fim, o ITB dos membros direito e esquerdo do paciente foi obtido por meio da razão entre a maior PAS do tornozelo direito e a maior PAS dos membros superiores, e a razão entre a maior PAS do tornozelo esquerdo e a maior PAS dos membros superiores, respectivamente^17,21^ . Os resultados do ITB foram interpretados como normais quando o resultado ficou entre 0,9 e 1,3, fato que indica fluxo sanguíneo normal no membro inferior, sugestivo de calcificação arterial quando ITB ≥ 1,3 e relacionado com DAP quando ITB ≤ 0,9, indicando estenose arterial^13,17^ .

### Análise estatística

Os dados foram organizados em estatística descritiva e exibidos em tabela. O teste qui-quadrado foi utilizado para avaliar a relação entre a presença de alterações arteriais (DAP ou calcificação arterial) e variáveis clínicas e sociodemográficas. Valor de p < 0,05 foi considerado estatisticamente significativo.

### Aspectos éticos

Como esta pesquisa envolve seres humanos, o projeto foi aprovado pelo Comitê de Ética em Pesquisa (CEP), sob o CAAE nº 58588522.3.0000.5666 e executado de acordo com a Resolução nº 510/2016 do Conselho Nacional de Saúde. Além disso, foi solicitada a autorização e a colaboração da CLIRENAL.

## RESULTADOS

Foram examinados 85 pacientes em hemodiálise. Desses, 21 (24,7%) apresentaram ITB ≤ 0,9, compatível com DAP (média: 0,7), e 19 (22,4%) apresentaram ITB ≥ 1,3, compatível com calcificação arterial (média: 1,5).

Constatou-se que a DAP apresentou maior prevalência em pacientes do sexo feminino (66,7%, n = 14), com idade > 60 anos (76,2%, n = 16), IMC eutrófico (52,4%, n = 11), com menos de 12 meses de hemodiálise (61,9%, n = 13) e sedentários (85,7%, n = 18). A distribuição dos casos foi homogênea quanto à etnia [parda 38,1% (n = 8), negra 33,3% (n = 7) e branca 28,6% (n = 6)].

A calcificação arterial foi mais prevalente nos indivíduos do sexo masculino (63,2%, n = 12), com idade > 60 anos (52,6%, n = 10), IMC eutrófico (68,4%, n = 13), com menos de 12 meses de hemodiálise (57,9%, n = 11) e sedentários (78,9%, n = 15). A distribuição dos casos foi homogênea quanto à etnia [parda 26,3% (n = 5), negra 36,8% (n = 7) e branca 36,8% (n = 7)].

Entre os pacientes com alterações arteriais (DAP ou calcificação arterial), o consumo de álcool foi mais prevalente entre os participantes com calcificação arterial (63,2%, n = 12), enquanto os tabagistas representaram a maioria dos casos de DAP (57,1%, n = 12). As comorbidades mais prevalentes foram hipertensão arterial (> 84,2%) e DM (> 52,6%).

A presença de sintomatologia, caracterizada por claudicação intermitente, alterações tróficas em extremidades e feridas ou úlceras que não cicatrizavam, foi maior no grupo com ITB compatível com DAP (p = 0,03). A presença de DM (p = 0,02) e idade mais avançada (p = 0,01) configuraram maior chance de DAP (Tabela 1).

**Tabela 1:** Perfil clínico e sociodemográfico dos participantes incluídos no estudo (N = 85).

**Variáveis**	**Frequência n (%)**	**ITB n (%)**			
		**Calcificação arterial**	**p**	**DAP**	**p**
Sexo feminino	45 (52,9)	7 (36,8)	0,11	14 (66,7)	0,15
Idade > 60 anos	45 (52,9)	10 (52,6)	0,66	16 (76,2)	**0,01**
Sobrepeso/obesidade	29 (34,1)	6 (31,6)	0,79	10 (47,6)	0,13
Etnia negra	31 (36,5)	7 (36,8)	0,97	7 (33,3)	0,73
Tempo de hemodiálise > 12 meses	31 (36,5)	8 (42,1)	0,56	8 (38,1)	0,86
Sedentarismo	71 (83,5)	15 (78,9)	0,54	18 (85,7)	0,76
Uso de álcool	45 (52,9)	12 (63,2)	0,31	9 (42,9)	0,29
Tabagismo	39 (45,9)	9 (47,4)	0,88	12 (57,1)	0,24
Hipertensão arterial	72 (84,7)	16 (84,2)	0,95	19 (90,5)	0,40
Histórico familiar de DCV	39 (45,9)	7 (36,8)	0,37	11 (52,4)	0,49
DM	35 (41,2)	10 (52,6)	0,25	13 (61,9)	**0,03**
DCV	32 (37,6)	8 (42,1)	0,65	8 (38,1)	0,96
Dislipidemia	26 (30,6)	7 (36,8)	0,50	9 (42,9)	0,16
Presença de sinais e sintomas compatíveis com DAP	32 (37,6)	4 (21,1)	0,09	12 (57,1)	**0,03**
Total	85 (100,0)	19 (22,4)		21 (24,7)	

DM = diabetes melito; DCV = doença cardiovascular; DAP = doença arterial periférica; ITB = índice tornozelo-braquial. Análise estatística realizada pelo teste qui-quadrado.

## DISCUSSÃO

Verificou-se prevalência de alterações arteriais (DAP ou calcificação arterial) em quase metade dos pacientes em hemodiálise. Os principais fatores de risco associados à DAP foram DM e idade avançada. Esses achados ratificam a necessidade e a importância da mensuração do ITB em pacientes com DRC, em virtude da suscetibilidade a fatores de risco cardiovasculares cumulativos e da demanda por estratégias diagnósticas rápidas, eficientes e integradas à rotina clínica.

Evidenciou-se que 24,7% da população analisada com DRC apresentou ITB compatível com DAP. Essa prevalência é condizente com os achados de um estudo multicêntrico espanhol realizado com 2.445 pacientes (28%)^28^ ; no entanto, um estudo brasileiro denotou prevalência menor (11%)^30^ .

A DAP é descrita como mais prevalente na população com DRC do que na população em geral^5^ . A DAP é uma vasculopatia comumente associada à DRC pelo fato de ambas compartilharem fatores de risco em suas bases etiológicas, e o diagnóstico precoce é importante para evitar possíveis complicações^31^ . Apesar de sinais e sintomas estarem presentes na maioria dos pacientes diagnosticados com DAP, as manifestações clínicas são inespecíficas e podem decorrer de alterações osteomusculares, articulares ou venosas, decorrentes ou não da DRC. Desse modo, a ultrassonografia pode auxiliar na definição etiológica. A mensuração do ITB, como ferramenta de rastreio da DAP, pode auxiliar a minimizar desfechos adversos. Nesse contexto, o uso de tecnologias como a POCUS pode ser um importante aliado no diagnóstico precoce da DAP e na redução de suas complicações^32-34^ .

Constatou-se que a maioria dos pacientes com DAP era tabagista. Apesar do tabagismo ser um importante fator de risco para DAP, não houve associação significativa com a presença da doença, provavelmente em razão do número limitado de pacientes participantes da pesquisa.

O DM foi associado à presença de DAP, corroborando estudos anteriores^6,28,30^ . Esse resultado reafirma a influência importante do DM na gênese da DAP, uma vez que o DM gera inflamação vascular e dano epitelial, além de vasoconstrição associada à ativação plaquetária^35^ . Apesar de outras comorbidades, como hipertensão arterial, obesidade, doença cerebrovascular e dislipidemia, também serem fatores de risco para DAP, não foi evidenciada associação significativa na população estudada.

A terapia hemodialítica gera alterações vasculares a longo prazo^36^ , por isso, espera-se que indivíduos submetidos a esse tratamento por mais tempo apresentem maior prevalência de DAP. No entanto, não foi observada relação entre tempo de diálise e incidência de DAP, achado também demonstrado em estudos anteriores^30,37^ .

Evidenciou-se prevalência de 22,4% de exames com ITB ≥ 1,3, compatível com calcificação arterial, nos pacientes avaliados neste estudo. Em pesquisa prévia, Ramos et al.^30^ evidenciaram que até 46,2% dos pacientes em terapia hemodialítica apresentam calcificação vascular. Tal diferença possivelmente se deve ao tempo de hemodiálise ao qual os indivíduos foram expostos^38^ . A calcificação arterial é uma importante complicação da DRC, em decorrência do distúrbio do equilíbrio mineral e ósseo, e confere maior risco de mortalidade aos seus portadores^38,39^ .

A calcificação arterial está relacionada ao aumento de rigidez vascular e à presença de artérias não compressíveis. Dessa forma, proporciona maior espessamento das artérias renais, maior prevalência de lesão microvascular renal e, como consequência, redução da taxa de filtração glomerular. Ademais, um ITB elevado, por exemplo, pode levar à sobrecarga hemodinâmica, à disfunção endotelial e à hipertrofia da massa do ventrículo esquerdo, o que aumenta o risco de eventos cardiovasculares, especialmente em pacientes com DRC^40^ . Assim como na DAP, a calcificação arterial pode ser investigada clinicamente por meio da mensuração do ITB.

A POCUS viabiliza a mensuração do ITB, com visualização da artéria, facilitando a definição da PAS para o ITB. Esse aspecto ganha ainda mais importância quando se trata de pacientes que frequentemente apresentam alterações arteriais, como calcificação, e são avaliados em contexto coletivo, à beira leito, em locais com excesso de ruídos. Além disso, a POCUS permite que profissionais capacitados avaliem de forma mais abrangente a DAP, orientando a estratificação da doença e o manejo mais adequado do caso. Ainda, a POCUS é uma ferramenta útil na avaliação de outros órgãos e sistemas importantes no contexto da DRC e da terapia hemodialítica, características evidenciadas também por outros autores^21,22,34^ .

É importante destacar que o método POCUS possui limitações, visto que demanda necessidade de experiência e treinamento especializado, bem como mais tempo para realização, quando analisado em prática de larga escala^32,34^ . Quanto às limitações do estudo, observa-se que valores de ITB ≥ 1,3 podem subestimar a prevalência de DAP, uma vez que o exame pode ser falso-negativo nesse público. Outra limitação diz respeito ao grande número de pacientes com fístulas arteriovenosas como acesso vascular para diálise, o que limitou a população do estudo e o número final de participantes em cada grupo avaliado, o que não permitiu análises estatísticas mais robustas.

Por se tratar de um estudo transversal, analítico e observacional, não foi possível estabelecer relações de causa e efeito entre os fatores de risco e as alterações arteriais. Ademais, a pesquisa foi realizada em um único centro, o que limita a generalização dos resultados a outras populações de pacientes em hemodiálise ou a diferentes contextos geográficos e de saúde.

## CONCLUSÃO

Identificou-se, por meio da POCUS, prevalência de 24,7% de alterações arteriais compatíveis com DAP e de 22,4% compatíveis com calcificação arterial na amostra de pacientes com DRC em hemodiálise. Adicionalmente, evidenciou-se que a idade avançada e o diagnóstico de DM foram os principais fatores associados à presença de DAP nesses indivíduos. Conclui-se que a POCUS se mostrou um método exequível para mensuração do ITB e detecção de alterações arteriais à beira leito no centro de estudo, reforçando a necessidade de monitoramento vascular contínuo nessa população, visto que quase metade dos indivíduos apresentou comprometimento vascular.
